# Editorial: NeuroDesign in human-robot interaction: the making of engaging HRI technology your brain can’t resist

**DOI:** 10.3389/frobt.2025.1699371

**Published:** 2025-10-30

**Authors:** Ker-Jiun Wang, Ramana Vinjamuri, Maryam Alimardani, Tharun Kumar Reddy, Zhi-Hong Mao

**Affiliations:** 1 Department of Electrical and Computer Engineering, University of Pittsburgh, Pittsburgh, PA, United States; 2 Department of Computer Science and Electrical Engineering, University of Maryland, Baltimore County, Baltimore, MD, United States; 3 Department of Computer Science, Vrije Universiteit Amsterdam, Amsterdam, Netherlands; 4 Department of Electronics and Communication Engineering, Indian Institute of Technology Roorkee, Roorkee, India

**Keywords:** neurodesign, human-robot interaction (HRI), brain-machine interface (BMI), neuroadaptive interfaces, neuroergonomics, physiological computing, human-centered AI, wearable robotics

## 1 Introduction

NeuroDesign in Human-Robot Interaction (HRI) is an emerging field that asks a simple but transformative question: What if we design robots with our human brain in mind? Unlike traditional approaches that focus primarily on functional or task-oriented measures, NeuroDesign integrates insights from neuroscience, cognitive and behavioral psychology, robotics, AI, and interaction design to create human–robot systems that are neurologically intuitive, emotionally resonant, and cognitively and ergonomically aligned with how we think and move. The goal is not only to optimize performance but also to design experiences that are natural and intuitive to our brain and body.

The design approaches focus on coherence across all levels of the human–robot system: from the robot’s physical form and motion patterns to its inner control logic, AI decision-making, and multimodal sensor integration. Whether a robot is synchronizing with a user’s muscle activity, regulating its behavior based on mental workload, or reacting to affective signals with haptic and voice feedback, NeuroDesign considers a holistic view of co-adaptation between humans and machines. The objective is not merely usability, but engagement—just as what this Research Topic’s title describes: The making of engaging HRI technology your brain can’t resist.

Basically, NeuroDesign involves both cognitive human-robot interaction (cHRI) (Mutlu et al., 2016) and physical human-robot interaction (pHRI) (Haddadin and Croft, 2016). It includes four fundamental modes of brain-body-robot interaction, each a bidirectional loop between human and machine: (1) Human Brain ⟷ Robot Brain Interaction Loop (cHRI) represents cognitive interaction between user intent, as decoded from neural or attentional signals, and robotic decision-making, which provides feedback through visual or auditory cues. (2) Human Brain ⟷ Robot Body Interaction Loop (cHRI) involves thought-controlled interfaces to guide robotic motion, with robots providing feedback in the form of expressive cues from their bodies. (3) Robot Brain ⟷ Human Body Interaction Loop (cHRI) places adaptive robotic intelligence in direct interface with human physiology, shaping experience through haptics, visual feedback, or affect-aware signals. And the (4) Human Body ⟷ Robot Body Interaction Loop (pHRI) encompasses physical synchrony, where muscle activity, joint motion, and biomechanics drive collaboration through wearable robots, cobots, or co-manipulation tasks. These loops are facilitated by multi-modal sensing (e.g., EEG, EMG, IMU, eye gaze, speech, skin conductance) and require meticulous integration of hardware, software, and user experience design.

## 2 Implementing the loops: contributions from this research topic

### 2.1 Human Brain ⟷ Robot Brain Interaction Loop (cHRI)

The papers in this Research Topic demonstrate how such loops can be implemented in practice. For example, Arulkumaran et al. demonstrate how visual and auditory P300 EEG interfaces can influence robot task control according to individual attentional preferences, illustrating the Human Brain ⟷ Robot Brain Interaction Loop. Similarly, Vieira et al. demonstrate that action anticipation from EEG can predict user movement hundreds of milliseconds before its onset, enabling robots to proactively coordinate with human intention. Both studies highlight how robot intelligence can “read ahead” of the body by decoding neural signals, forming a true cognitive-to-cognitive collaboration.

### 2.2 Human Brain ⟷ Robot Body Interaction Loop (cHRI)

In the Human Brain ⟷ Robot Body Interaction Loop, Molnar et al. illustrate how personalized teleoperation mappings derived from trajectory clustering align with users’ internal mental models, making robot motion feel immediately intuitive. Chenais and Görgen extend this principle to clinical contexts, where immersive XR systems translate thought-based interactions into robotic or virtual actions, while avatars and visual feedback provide embodied channels of communication between the user and the robot.

### 2.3 Robot Brain ⟷ Human Body Interaction Loop (cHRI)

The Robot Brain ⟷ Human Body Interaction Loop is prominently highlighted in the review by Pilaciński et al., who propose integrating human activity recognition and brain–machine interfaces so that collaborative robots can infer not just what the body is doing, but also what the brain is going to do. Chenais and Görgen’s review also contributes to this discussion, describing how XR systems can adapt feedback to a user’s affective or physiological state in real-time—showing how robots can reshape bodily and emotional responses through adaptive intelligence.

### 2.4 Human Body ⟷ Robot Body Interaction Loop (pHRI)

Finally, the Human Body ⟷ Robot Body Interaction Loop is exemplified by Mehta et al., who introduce a neural efficiency metric to measure how exoskeletons alter both biomechanics and cognitive load during industrial lifting activities. This work quantifies how wearable robots affect not only muscles and joints but also brain adaptation and efficiency. Shaw et al. also explore human–human haptic co-manipulation, extracting communicative primitives of force, motion, and degrees of freedom. Their work provides design guidelines for robots that physically collaborate with humans as naturally as human partners do.

Across the papers in this Research Topic, several unifying design principles emerge. First, HRI systems should adapt to internal cognitive models, as shown by personalized mappings (Molnar et al.) and sensory modality preferences (Arulkumaran et al.). Second, robust intent decoding requires the fusion of behavior and brain, aligning bodily action with neural precursors (Pilaciński et al.). Third, systems can exploit anticipation through EEG signals that reveal intent before motion begins (Vieira et al.). Fourth, metrics such as neural efficiency highlight hidden cognitive loads of assistive devices (Mehta et al.), broadening design goals beyond physical mechanics. Fifth, haptics can be treated as a communication channel with its own rules (Shaw et al.). Finally, XR applications (Chenais and Görgen) emphasize the importance of bridging laboratory insight with clinical practice, ensuring translational impact.

While the “Four-Loop” framework effectively captures the essence of dyadic human–robot interaction, it can also be extended to more complex scenarios involving multiple humans, multiple robots, and broader social or organizational dynamics. These expansions bring forth important ethical questions: How do we ensure transparency, agency, and inclusiveness in systems that adapt based on brain and body signals? NeuroDesign provides a cohesive lens through which to explore and address these frontiers.

## 3 The future of NeuroDesign: brain and body as active co-designers

Looking ahead, NeuroDesign invites us to reimagine the role of the brain and body in the design process—not as passive endpoints of interaction, but as active co-designers. The future of HRI will not be defined solely by smarter algorithms or faster actuators, but by how seamlessly robotic systems become integrated into their users’ cognitive and physical lives. Across these seven papers, we see advances in personalization, multimodal sensing, predictive modeling, and physical communication that lay the groundwork for HRI technologies that are not only usable but also engaging in brain-mediated experiences. The four loops also provide a blueprint for designing systems in these papers: teleoperation that feels cognitively seamless, interfaces that adapt to neural diversity, robots that anticipate and modulate bodily states, and wearable systems that physically synchronize like trusted partners. NeuroDesign’s emphasis on engagement, rather than mere usability, marks a conceptual shift with the potential to spark novel research trajectories in HRI.

To realize this vision, several key challenges also emerge as priorities to be addressed: (i) achieving real-time multimodal fusion of neural, physiological, and behavioral signals for adaptive interactions; (ii) developing personalized cognitive and motor models that reflect the neural and bodily diversity of users; and (iii) enhancing explainability and trust in adaptive autonomous systems, ensuring that brain- and body-driven adaptations remain transparent and reliable. Addressing these challenges will help transform the NeuroDesign paradigm into a tangible, actionable roadmap for the research community.

## 4 Conclusion

This Research Topic’s collection, therefore, provides a guide to that future. These studies shape a future generation of wearable systems, BCIs, cobots, and XR platforms that put the brain—not just performance—at design’s core. Together, they bring us closer to a future where robots are not just functional and efficient—but felt, understood, and trusted by the people they serve.

## References

[B1] Haddadin S. Croft E. (2016). “Physical human–robot interaction,” in Springer handbook of robotics. 2nd edn. (Cham: Springer), 1835–1874. 10.1007/978-3-319-32552-1_69

[B2] Mutlu B. Roy N. Šabanović S. (2016). “Cognitive human–robot interaction,” in Springer handbook of robotics. 2nd edn. (Cham: Springer), 1907–1934. 10.1007/978-3-319-32552-1_71

